# An Editorial for Mechanical Performance and Microstructural Characterization of Light Alloys (2nd Edition)

**DOI:** 10.3390/ma18235330

**Published:** 2025-11-26

**Authors:** Qinghuan Huo

**Affiliations:** School of Materials Science and Engineering, Central South University, Changsha 410083, China; huoqinghuan@csu.edu.cn

Although studies on the mechanical performance of light metals and their alloys have been carried out all over the world [1,2,3,4], difficult challenges still persist in further improving their strength. As the most widely applied light alloys, aluminum alloys possess the ideal combination of strength and ductility [5,6]. However, their welded joints always demonstrate a much lower strength compared with their base materials, owing to their high stack fault energy and hard recrystallization potential [7,8]. Magnesium and its alloys, as the lightest structural materials, present different drawbacks, displaying poor tensile ductility and corrosion resistance [9,10,11]. Titanium alloys with different phase combinations show the ideal corrosion resistance and yield strength, but their ultimate tensile strength is still far below the strength of steel [12,13]. With the advances in additive manufacturing and machine learning [14,15,16], some new types of titanium alloys and titanium-based composites have demonstrated much better mechanical properties, such as an extremely high strength close to that of steel, a long fatigue life, and even lighter total weights. Nonetheless, difficulties remain regarding alloys’ mechanical properties and manufacturing methods which must be solved in the near future [17,18]. For the sake of overcoming the non-ideal mechanical performance of light alloys, novel processing methods and new composition designs must be introduced using various characterization trials to study microstructures in depth [19,20,21,22]. Namely, the recent progress in high-performance light alloys has been achieved by elucidating the close relationship between mechanical performance and microstructures.

At present, on the basis of the Special Issue entitled “Mechanical Performance and Microstructural Characterization of Light Alloys (1st Edition)”, this Second Edition aims to continue reporting on the most recent advances in research on the close relationship between mechanical performance and microstructure in light alloys. Also, advanced processing methods for aluminum, magnesium, titanium, and their alloys, and optimized characterization methods for microstructures are included. Specific highlights of the publications in this Second Edition are illustrated in the following two paragraphs, and detailed information for these publications can be found in the Section “List of Contributions”.

As a typical light alloy, the AA7075 aluminum alloy shows slow recrystallization and poor welding behavior. In light of this problem, Lacki et al. (contribution 1) performed friction stir welding to improve the local stored energy and to trigger the occurrence of recrystallization. Using the most suitable welding parameters, the joints showed the ideal mechanical performance. Wang et al. (contribution 2) used the radial forging method to induce local small straining on aluminum alloys. In this way, the stress distributions could be uniform and the mechanical strength could be successfully enhanced without any obvious ductility reduction. Similar mechanical responses have also been proposed by Yang et al. (contribution 3), who simplified stress directions into two orthometric ones and achieved an improved mechanical performance with aluminum tubes. In addition, some novel processing methods like additive manufacturing have also been developed by researchers. For example, Antolak-Dudka et al. (contribution 4) and Kluczyński et al. (contribution 5) focused on the preparation and characterization of Ti6Al4V alloys made by additive manufacturing. The authors put forward suitable parameters for enhancing the mechanical properties and corrosion resistance in detail. Similar trials have also been conducted for the surface treatments of magnesium alloys (contribution 6), which were confirmed to be successful.

Meanwhile, basic theories have been enhanced for aluminum, magnesium, titanium, and their alloys using microstructure characterizations and numerical simulations. Chen et al. (contribution 7) studied the effect of local straining on the deformation mode of titanium and renewed the interaction results of slip-on twinning. Sun et al. (contribution 8) combined molecular dynamics and microstructure observations and found novel phenomena in the twin boundaries of titanium alloys with different phases. Similar trials have also been performed by Zuo et al. (contribution 9) and Huang et al. (contribution 10). The former study provided one new simulation method and the latter study confirmed the applicability of numerical analysis on the metal-based composites. Furthermore, Zhang et al. (contribution 11) even extended the applicability to alloy material with liquid additions.

Based on the above-mentioned contributions on the mechanical performance and microstructure characterization of light alloys and metal-based composites, it is reasonable to expect improvements in the mechanical properties of these materials and continuous development in novel processing methods in the next few years. In particular, rapid formation technologies, short process methods, extremely improved mechanical strength, and cutting-edge microstructure characterizations will be pursued. Finally, we expect that this Second Edition will be an asset in related research fields; we hope that it is well received and that readers look forward to a Third Edition.
